# Nucleotidyl Cyclase Activity of Particulate Guanylyl Cyclase A: Comparison with Particulate Guanylyl Cyclases E and F, Soluble Guanylyl Cyclase and Bacterial Adenylyl Cyclases Cyaa and Edema Factor

**DOI:** 10.1371/journal.pone.0070223

**Published:** 2013-07-29

**Authors:** Kerstin Y. Beste, Corinna M. Spangler, Heike Burhenne, Karl-Wilhelm Koch, Yuequan Shen, Wei-Jen Tang, Volkhard Kaever, Roland Seifert

**Affiliations:** 1 Institute of Pharmacology, Hannover Medical School, Hannover, Germany; 2 Research Core Unit for Mass Spectrometry – Metabolomics, Hannover Medical School, Hannover, Germany; 3 Department of Biology and Environmental Sciences, Biochemistry Group, University of Oldenburg, Oldenburg, Germany; 4 College of Life Sciences, Nankai University, Tianjin, China; 5 Ben May Department for Cancer Research, University of Chicago, Chicago, Illinois, United States of America; German Research School for Simulation Science, Germany

## Abstract

Guanylyl cyclases (GCs) regulate many physiological processes by catalyzing the synthesis of the second messenger cGMP. The GC family consists of seven particulate GCs (pGCs) and a nitric oxide-activated soluble GC (sGC). Rat sGC α_1_β_1_ possesses much broader substrate specificity than previously assumed. Moreover, the exotoxins CyaA from *Bordetella pertussis* and edema factor (EF) from *Bacillus anthracis* possess nucleotidyl cyclase (NC) activity. pGC-A is a natriuretic peptide-activated homodimer with two catalytic sites that act cooperatively. Here, we studied the NC activity of rat pGC-A in membranes of stably transfected HEK293 cells using a highly sensitive and specific HPLC-MS/MS technique. GTP and ITP were effective, and ATP and XTP were only poor, pGC-A substrates. In contrast to sGC, pGC-A did not use CTP and UTP as substrates. pGC-E and pGC-F expressed in bovine rod outer segment membranes used only GTP as substrate. In intact HEK293 cells, pGC-A generated only cGMP. In contrast to pGCs, EF and CyaA showed very broad substrate-specificity. In conclusion, NCs exhibit different substrate-specificities, arguing against substrate-leakiness of enzymes and pointing to distinct physiological functions of cyclic purine and pyrimidine nucleotides.

## Introduction

Guanosine 3′,5′-cyclic monophosphate (cGMP) is a well-established second messenger that is involved in the regulation of many physiological processes like blood homeostasis, neurotransmission, intestinal secretion, and vision [1]. cGMP is generated from GTP by guanylyl cyclases (GCs) that are expressed in two isoforms: the soluble (sGC) and particulate or membrane-bound (pGC) form. Seven pGC isoforms have yet been found (pGC-A to pGC-G) [1]–[3]. The best described pGC is the natriuretic peptide (NP) receptor A (NRP1, pGC-A) that is activated by atrial NP (ANP) or less effectively by B-type NP (BNP) [1]. The receptor is a dimer consisting of an extracellular ligand binding domain and an intracellular domain which contains a transmembrane region, a juxtamembranous protein kinase homology domain (KHD), a hinge region, and an intracellular domain with the catalytically active GC site [1]. Recently, biochemical studies with pGC-A and pGC-B revealed that both isoforms possess separate and distinct catalytic and allosteric binding sites that bind GTP and ATP, respectively, under physiological conditions [4].

pGC-B is mainly expressed in vascular endothelial cells. pGC-B mediates vascular regeneration, cell growth, and endochondral ossification through binding of C-type natriuretic peptide and by activating cGMP-dependent protein kinase II [1]. pGC-C is expressed in the intestinal epithelium [1]. This pGC is activated by the peptides guanylin and uroguanylin, and bacterial heat-stable enterotoxins regulating electrolyte and water transport in the intestine, and epithelial cell growth and differentiation. pGC-D is located in the olfactory neuroepithelium playing an important role in odor recognition and modulating the sensitivity of sensory neurons [1].

Further members of the GC family are the isoforms pGC-E and pGC-F (ROS-GC-1 and ROS-GC-2). They play a key role in vertebrate phototransduction and are localized in the rod outer segment (ROS) [5]. pGC-E and pGC-F are regulated by neuronal calcium sensor proteins, named guanylyl cyclase-activating proteins (GCAPs). These regulatory proteins sense changes in the cytoplasmic Ca^2+^-concentration during illumination and activate ROS-GCs when the cytoplasmic Ca^2+^ decreases below the value in a dark-adapted cell [5]. The GCAP-dependent stimulation is inhibited by increasing Ca^2+^ [5].

Particulate fractions of sea urchin sperm expressing relatively high amounts of pGC-A were shown to generate inosine 3′,5′-cyclic monophosphate (cIMP) and 2′-deoxyguanosine 3′,5′-monophosphate beside cGMP in the presence of Mn^2+^ [6]. Additionally, the recombinantly expressed catalytic core of rat pGC-A synthesizes cGMP and 2′-deoxyguanosine 3′,5′-monophosphate in the presence of Mn^2+^ [7]. In the study by Thorpe et al. [7], it was also reported that with a radiometric method combined with thin-layer chromatography, ATP, CTP and UTP were not substrates for rat pGC-A, but no experimental data were documented in a Figure or a Table (data were reported only as data not shown). Moreover, the substitution of two amino acids in RetGC-1 converts the “guanylyl” cyclase into an “adenylyl” cyclase [8]. Rat sGC α_1_β_1_ generates other cyclic nucleotides like cXMP (xanthosine 3′,5′-cyclic monophosphate) and cCMP (cytidine 3′,5′-cyclic monophosphate) [9]. Since pGC-A and sGC exhibit ∼ 50% amino acid identity in the catalytic domain [10], here, we studied the substrate-specificity of pGC-A as well as pGC-E and pGC-F of bovine ROS. We used HPLC-MS/MS for determination of kinetic parameters for generation of the following seven 3′,5′-cyclic monophosphates (cNMPs): adenosine 3′,5′-cyclic monophosphate (cAMP), cCMP, cGMP, cIMP, thymidine 3′,5′-cyclic monophosphate (cTMP), uridine 3′,5′-cyclic monophosphate (cUMP), cXMP [9]. Additionally, we systematically analyzed the substrate-specificity of the adenylyl cyclase (AC) toxins CyaA from *Bordetella pertussis* and edema factor (EF) from *Bacillus anthracis* by HPLC-MS/MS because both toxins generate cCMP and cUMP as assessed by radiometric and HPLC-based methods [11]. The comparison of EF with pGC-A is also important because the toxin, like pGC-A from sea urchin sperm generates cIMP [6], [11], [12]. Furthermore, CyaA and EF are potently inhibited by various purine- and pyrimidine-substituted 2′(3′)-O-(N-methylanthraniloyl)-nucleotides [13], [14] indicating a high flexibility of their catalytic sites and pointing to potentially broad substrate-specificity. Our studies reveal very distinct substrate-specificities of various NCs, ranging from very broad (bacterial AC toxins) to relatively narrow (pGCs).

## Materials and Methods

### Materials

NTPs of adenine (2·Na·ATP, ≥99%), uracil (3·Na·UTP, ≥96%), hypoxanthine (3·Na·ITP, 95–97%) and guanine (GTP, ≥95%) as well as Na·cAMP, magnesium chloride, sodium chloride, EDTA, HEPES, MOPS, potassium chloride, 3-isobutyl-1-methylxanthine (IBMX), leupeptin, aprotinin, pepstatin, zaprinast, phosphocreatine, creatine phosphokinase, ANP, and bovine serum albumin (BSA) were purchased from Sigma-Aldrich (Seelze, Germany). Cytidine 5′-triphosphate (CTP, >99%), xanthosine 5′-triphosphate (XTP, >95%), and thymidine 5′-triphosphate (dTTP, >95%) were obtained from Jena Bioscience (Jena, Germany). All nucleoside 5′-triphosphates (NTPs) were prepared as 10–100 mM stock solutions with an equimolar concentration of a divalent cation (Me^2+^), either Mn^2+^ or Mg^2+^ and stored at −20°C. For preparation of NTP stock solutions, chloride salts of divalent cations were used. Glycerol was purchased from Serva (Heidelberg, Germany). Tris(hydroxylmethyl)-aminomethane hydrochloride (TRIS•HCl) was purchased from Merck (Darmstadt, Germany). cGMP, cUMP, cIMP, cCMP, cXMP, and cTMP (cNMP·Na) were supplied by Biolog (Bremen, Germany). Manganese chloride tetrahydrate, hydrochloric acid, and ammonium acetate were purchased from Fluka (Buchs, Switzerland). Acetonitrile, methanol, and water were supplied by Baker (Deventer, The Netherlands) and acetic acid was purchased from Riedel-de Haën (Seelze, Germany). [α-^32^P]GTP (3,000 Ci/mmol) was purchased from Hartmann Analytic (Braunschweig, Germany). Aluminium oxide N Super 1 was purchased from MP Biomedicals (Eschwege, Germany). Tenofovir was obtained through the National Institute of Health AIDS Research and Reference Program, Division of AIDS (Bethesda, MD, USA).

### Membrane Preparation of HEK293 Pgc-A Cells

HEK293 cells stably expressing FLAG-tagged rat pGC-A were kindly provided by Dr. Michaela Kuhn (Institute of Cardiovascular Physiology, University of Würzburg, Germany) and grown in DMEM high glucose 4.5 g/L (PAA, Cölbe, Germany) supplemented with penicillin at 100 U/mL, streptomycin at 0.1 mg/mL, L-glutamine at 0.292 g/L (PAA) and 10% (v/v) FBS (Lonza, Basel, Switzerland) up to 70–80% confluency [15]. For membrane preparations cells were washed twice in 1× Dulbeccós PBS (PAA) and re-suspended in homogenization buffer (50 mM HEPES pH 7.4, 100 mM NaCl, 1 mM EDTA, 10% (v/v) glycerol, 2 µg/mL aprotinin, 5 µg/mL pepstatin, and 2 µg/mL leupeptin). Cells were lysed using a nitrogen cavitation bomb (Parr, Moline, IL, USA) after incubation at 100 bar for 30 min. Lysate was centrifuged for 5 min at 500 × g and 4°C to remove cell nuclei. Subsequently, the supernatant fluid was centrifuged for 20 min at 30,000 × g and the pellet was re-suspended in 1 mL homogenization buffer using a 22G-needle. Membrane preparations were aliquoted in 10–100 µL batches and stored at −80°C until analysis. Protein concentrations of membrane preparations were determined by the Bradford protein quantitation method.

### NC Assay for pGC-A in HEK293 Cell Membranes

Membrane preparations of HEK293 cells stably overexpressing rat pGC-A (10–80 µg of protein per tube) were incubated in a total volume of 100 µL at 37°C in the presence of incubation buffer, 2–7,500 µM NTP/Me^2+^, plus additional 4 mM of the corresponding MeCl_2_. The latter MeCl_2_ addition is not anymore indicated in legends for the sake of brevity. In preliminary studies (Figs. S1 and S2), we conducted time course experiments with 200 µM NTP/Me^2+^ to identify linear conditions for determination of precise enzyme kinetics and to allow comparison with the data previously published for sGC [9]. Kinetic studies were then conducted under linear conditions previously identified in the time course experiments shown in Figs. S1 and S2. Reaction conditions for kinetic studies ensured that no substrate depletion occurred with >90% of the substrate still being present. Assays were stopped by heating for 10 min at 95°C. After cooling, mixtures were diluted by 200 µL of a solution consisting of 97/3 (v/v) water/methanol containing 50 mM ammonium acetate, 0.1% (v/v) acetic acid, and 100 ng/mL tenofovir. Denatured protein was precipitated by centrifugation for 10 min at 20,000 × g. Quantitation via HLPC-MS/MS was performed as described [9].

### NC Assay for pGC-A in Intact HEK293 Cells

HEK293 cells stably overexpressing rat pGC-A were seeded in a 6-well-plate with 5·10^5^ cells per well for 24 h in DMEM high glucose with 10% (v/v) FBS and 200 µg/mL L-glutamine, 100 U/mL penicillin, and 0.1 mg/mL streptomycin. The next day, cells were pre-incubated for 10 min with IBMX (100 µM) followed by stimulation with 1 µM ANP for various times. To stop stimulation and cell metabolism, medium was removed and 300 µL extraction solution consisting of acetonitrile/methanol/water (2∶2:1, v/v/v)) and 25 ng/mL tenofovir was added. Cell suspension was heated for 20 min at 95°C and centrifuged at 20,000 × g for 10 min to remove protein. Supernatant fluid was evaporated completely under nitrogen atmosphere at 40°C. Residue was resolved in 150 µL water and analyzed by HPLC-MS/MS as described in Ref. 9 except the fact that separation was performed on an Aglient 1100 series (Waldbronn, Germany) and for detection the more sensitive QTrap 5500 triple quadrupole mass spectrometer (ABSCIEX, Foster City, CA, USA) was used which is, according to our own experiments, up to 5-fold more sensitive than the mass spectrometer applied in the previous study. Parameters of HPLC-MS/MS fragments are documented in Table 1. Ion source settings and collision gas pressure were manually optimized regarding ion source voltage, ion source temperature, nebulizer gas, and curtain gas (ion source voltage of 5,500 V; ion source temperature of 600°C; curtain gas of 30 psi; collisionally activated dissociation gas of 9 psi). Chromatographic data were collected and analyzed with Analyst 1.5.1 (ABSCIEX). Quantitation was performed with nitrogen as collision gas. For determination of protein concentration, cell pellets of extraction procedure were dried at room temperature and resolved in 0.1 M NaOH at 95°C for 20 min. Ten microliters of protein solution were removed for quantitation of protein concentration by means of bicinchoninic acid protein assay.

**Table 1:** Parameters for the detection and quantitation of cNMPs and MS standard tenofovir by HLPC-MS/MS.

	cAMP	cCMP	cGMP	cIMP	cTMP	cUMP	cXMP	tenofovir
[M+H]^+^ [m/z]	330.0	306.0	346.0	331.0	305.0	307.0	346.8	288.0
Quantifier [m/z]	135.9	112.0	151.9	136.9	80.9	96.9	153.0	176.0
Qualifier [m/z]	312.0	95.1	135.0	110.1	126.9	112.9	136.0	270.0
Ratio quantifier/qualifier	1∶5.2	1∶4.0	1∶2.5	1∶7.2	1∶2.9	1∶1.9	1∶2.9	1∶2.2
Retention time [min]	6.6	4.0	5.5	5.5	5.8	5.0	2.8	5.8

Protonated molecule mass [M+H]^+^, HPLC retention time, MS/MS fragments of quantifier and qualifier as well as the ratio between quantifier and qualifier for quantitation of cNMP via HPLC-MS/MS.

### NC Assay with ROS Membrane Preparations

Bovine ROS was prepared as described in Ref. [16] under very dim red light using sucrose density centrifugation in the presence of 115 mM NaCl, 2.5 mM KCl, 1 mM MgCl_2_, 10 mM HEPES/KOH pH 7.5 and 1 mM DTT. For GC assays ROS membranes (81 µg rhodopsin per tube) were incubated for 5 min at 30°C with 1 mM GTP/Mg^2+^, 1 mM UTP/Mg^2+^ or 1 mM UTP/Mg^2+^. Buffer contained 30 mM MOPS pH 7.2, 60 mM KCl, 4 mM NaCl, 3.5 mM MgCl_2_, 0.3 mM ATP, 0.16 mM zaprinast, 1 mM DTT, and 2 mM EGTA or 2 mM CaCl_2_, as indicated. Reactions were stopped by heating at 95°C for 10 min and analyzed as described in [17]. In brief, concentrations of cGMP, cCMP, cUMP, GMP, CMP, and UMP were determined by a LC/MS system consisting of a binary HPLC pump (1100 Series, Agilent, Waldbronn, Germany) directly coupled to a single quadrupole mass spectrometer (LC-MSD SL, Agilent) operating in negative ion mode. Fifty µL of sample solution were loaded onto a Hypercarb column (30 × 4.6 mm, 5 µm particle size) (ThermoFisher, Dreieich, Germany) preceded by a column saver (2 µm, Supelco Analytical) and a C_18_ security guard (AJO-4286, Phenomenex). Compounds were separated by means of a linear gradient from 96% (v/v) eluent A consisting of 10 mM ammonium acetate (pH 10.0) up to 60% eluent B (acetonitrile) within 8 min and a flow rate of 0.4 mL/min. Subsequently, re-equilibration to 96% eluent A was conducted for 5 min. The following deprotonated molecule masses ([M-H]^-^) and retention times were determined: cGMP: 344.1 m/z, 7.6 min; cCMP: 304.1 m/z, 6.5 min; cUMP: 305.1 m/z, 6.1 min; GMP: 362.1 m/z, 5.4 min; CMP: 322.1 m/z, 4.2 min; UMP: 323.1 m/z, 4.1 min; internal standard tenofovir: 286.1 m/z, 5.9 min.

### NC Activity Assay with Purified Cyaa and EF

*E. coli* cells were transfected with plasmid pProExH6-EF and pExCyaA-N. Full-length EF and the catalytic domain of CyaA-N (amino acids 1 to 373) were purified from *E. coli* [18]. CaM was purified from calf brain [19]. The activity of the extracted enzymes was tested by assays with [α-^32^P]ATP [13]. [α-^32^P]ATP (3,000 Ci/mmol) was purchased from Perkin Elmer Life Sciences (Boston, MA, USA). For Michaelis-Menten kinetics of EF and CyaA-N with the substrate ATP 100 pM enzyme was used, whereas 100 nM of AC toxins was applied when determining k_cat_ and K_M_ of other NTPs. The NTP/Mg^2+^ concentrations were varied between 5 µM and 1 mM. The CaM concentration was adjusted to a stoichiometry of 1∶10 for AC toxin to CaM. The assay tubes contained final concentrations of 5 mM Mg^2+^, 10 µM Ca^2+^, 10 mM TRIS·HCl pH 7.5, and 0.1 wt% BSA. The temperature for the assay was set to 37°C and reaction times were varied according to the NTP/Mg^2+^ used (GTP/Mg^2+^: 30 min; TTP/Mg^2+^: 60 min; CTP/Mg^2+^, UTP/Mg^2+^: 10 min; ATP/Mg^2+^: 5 min; XTP/Mg^2+^, ITP/Mg^2+^: 90 min). The total volume was 50 µL per tube. The reaction was stopped by heat-inactivation at 95°C for 5 min. The precipitated proteins were removed by centrifugation. Fourty µL of the supernatant fluid was combined with 40 µL of internal standard (IS) solution. As internal standard cXMP was used at a concentration of 200 ng/mL when determining the turnover of all NTPs except for XTP/Mg^2+^. When the Michaelis-Menten kinetic of XTP/Mg^2+^ was determined, 200 ng/mL cIMP was applied as internal standard. Quantitation of generated cNMPs was performed by HPLC-M/MS. The chromatographic separation of cAMP, cCMP, cUMP, cIMP, and cTMP and the internal standard cXMP was performed on a LC-10ADVP HPLC system (Shimadzu, Kyoto, Japan) equipped with a binary pump system. A combination of Supelco column saver (2.0 µm filter, Supelco Analytical, Bellafonte, CA, USA), security guard cartridge (C_18_, 4×2 mm) in an analytical guard holder KJO-4282 (Phenomenex, Aschaffenburg, Germany) and an analytical Nucleodur C_18_ Pyramid RP column (50×3 mm, 3 µm particle size, Macherey-Nagel, Düren, Germany) temperature controlled by a convenient HPLC column oven at 25°C was used. Eluent A consisted of 5 mM ammonium acetate and 0.1% (v/v) acetic acid in water and eluent B was methanol. The injection volume was 50 µL and the flow rate was 0.4 mL/min throughout the chromatographic run. Eluent A (100%) was used from 0 to 5 minutes followed by a linear gradient from 100% A to 70% A until 9 min. Eluent A (70%) was kept until 11 min and re-equilibration of the column at 100% A was achieved from 11 to 15 min. The retention times of analytes were as follows: cAMP: 9.7, cCMP: 5.0, cIMP: 8.7, cTMP: 8.8, cUMP: 7.9 and cXMP: 8.4 min. The analyte detection was performed on an API 2000 triple quadrupole mass spectrometer (Applied Biosystems) using selected reaction monitoring (SRM) analysis in positive ionization mode. The following SRM transitions (m/z) using a dwell time of 40 ms were detected: cAMP: +330/136; cCMP: +306/112; cIMP: +331/137; cTMP: +305/127; cUMP: +307/97; cXMP: +347/153. The mass spectrometer parameters were as follows: ion source voltage: 5500 V, temperature: 350°C, curtain gas: 40 psi, collisionally activated dissociation (CAD) gas: 5 psi.

### Statistics

Presented data are the mean with range of two independent experiments or the means ± standard error of the mean (SEM) of six independent experiments, as indicated. GraphPad Prism software version 5.01 (San Diego, CA, USA) was used for nonlinear regression and calculation of mean, SEM, s_0.5_, V_max_, *n*_Hill_, and IC_50_ values.

## Results

### Time-Courses of pGC-A

**Figure 1:**
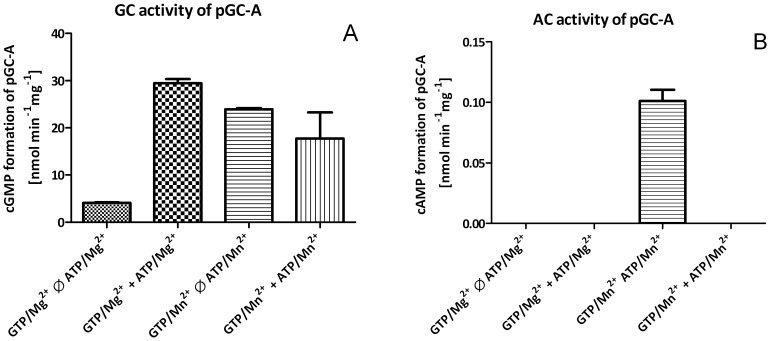
Effect of ATP on GC activity in membrane preparations of HEK293 cells stably overexpressing pGC-A. Membranes (10 µg of protein per tube) were incubated for 25 min at 37°C in the presence of 100 µM GTP/Me^2+^ with or without 500 µM ATP/Me^2+^ as indicated. Values represent the means ± SEM of three independent experiments. Please note the different scales of the y-axes in both panels.

In order to identify suitable experimental conditions for determination of enzyme kinetics, we first investigated the time-dependent generation of cNMPs (Figs. S1 and S2). Membranes (10–80 µg protein per tube) from HEK cells stably expressing pGC-A were activated by 1 µM ANP in the presence of 200 µM NTP/Me^2+^ plus additional 4 mM MnCl_2_ or MgCl_2_, respectively, followed by incubation at 37°C for 2–100 min. In the presence of Mn^2+^ pGC-A showed a linear generation of cGMP for up to 100 min (Fig. S1A). However, when Mn^2+^ was replaced by Mg^2+^, we could hardly detect GC activity (Fig. 1) (10). Therefore, we added ATP/Mg^2+^ (500 µM) that reduces the Hill coefficient and decrease the K_M_ [4]. Under these conditions, a linear production of cGMP up to 15 min was observed (Fig. S2), with the GC activity being even higher than in the presence of Mn^2+^ (Fig. 1). We also examined the influence of ATP/Mn^2+^ (0.5 mM) on GC activity in the presence of additional 4 mM MnCl_2_. Under these conditions, an inhibitory effect on GC activity was reported [7], [20], [21]. Indeed, we observed a reduction of cGMP production, in parallel with cAMP generation (Fig. 1).

In the presence of Mn^2+^, pGC-A also generated cAMP and cXMP (Figs. S1C–D), although much lower catalytic activities than for cGMP formation were observed. Moreover, we observed cIMP formation in the presence of Mn^2+^ as well as in the presence of Mg^2+^ (Figs. S1 and S2). The formation rate in presence of Mn^2+^ was cIMP>> cGMP>> cAMP> cXMP. In contrast to sGC, no catalytic activity for the generation of cyclic pyrimidine nucleotides was observed (Figs. S1E and F). In the presence of Mg^2+^, the formation rate of cGMP was significantly higher than for cIMP (Figs. S2A and B), and generation of cAMP, cXMP, cCMP, cUMP, and cTMP could not be detected even after an incubation of 60 min (Fig. S2C). The detergent Triton X-100 largely enhanced the catalytic activity of pGC-A with ATP and GTP as substrates. However, Triton X-100 did not render CTP, TTP or UTP pGC-A substrates (Fig. S3).

### Enzyme kinetics of pGC-A in Membranes

**Figure 2:**
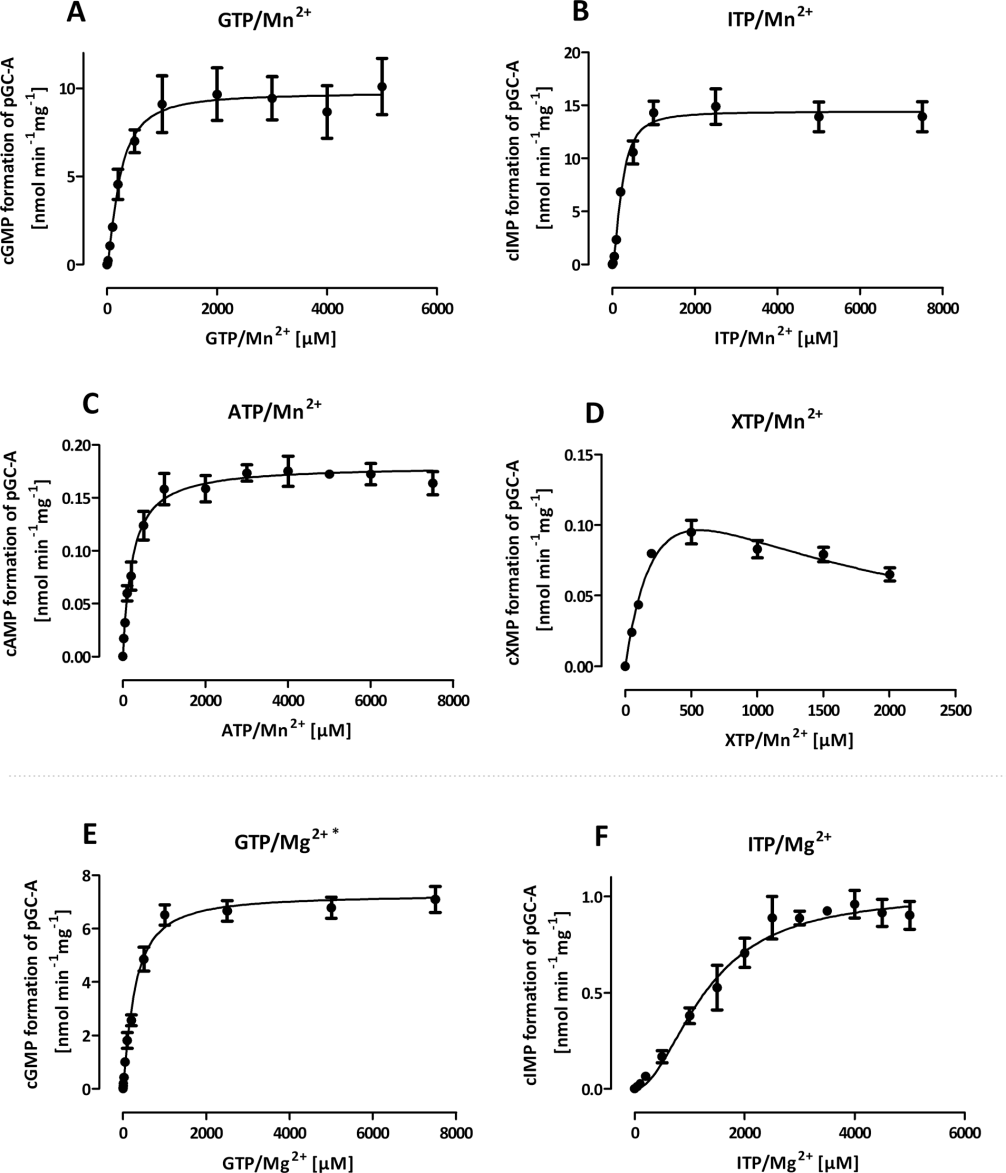
Kinetic analysis of nucleotidyl cyclase activity in membrane preparations of HEK293 cells stably overexpressing pGC-A. Membranes (10–80 µg protein per tube) were incubated with 2–7,500 µM NTP/Me^2+^ (XTP/Me^2+^: 2–2,000 µM) in the presence of 1 µM ANP at 37°C for 5–10 min. Values represent the mean ± SEM of six independent experiments. Except data of plot D, data were fitted using specific binding with Hill slope. Data of plot D were best fitted by substrate inhibition model. Please note the different scales of the x- and y-axes of all panels. *: only detectable in the presence of 500 µM ATP/Mg^2+^. Kinetic parameters are shown in Table 2.

**Figure 3:**
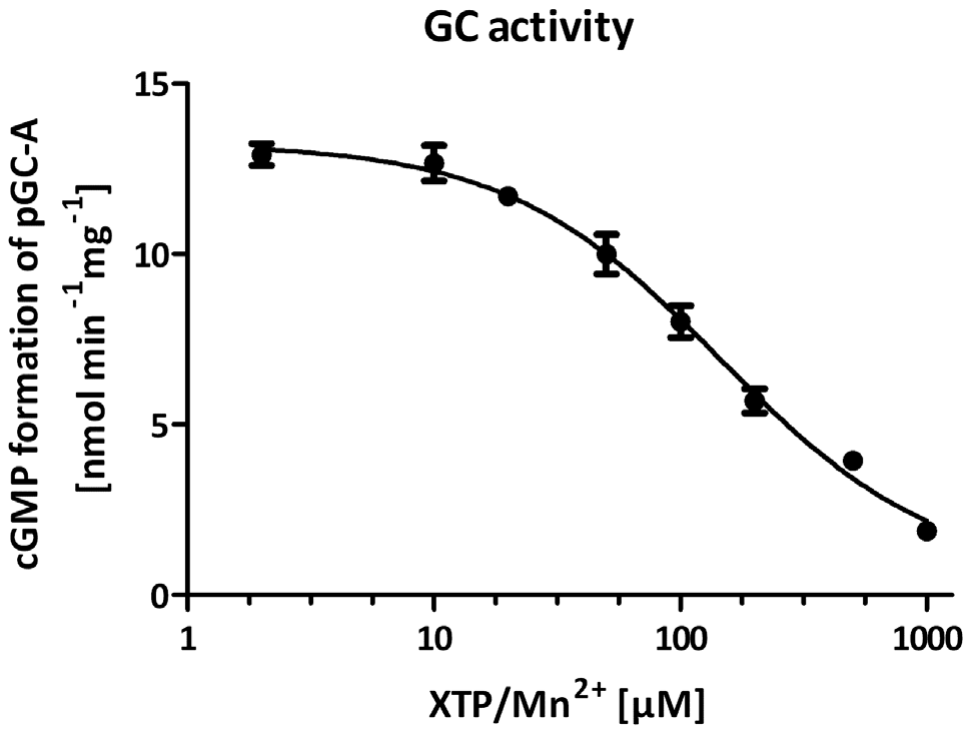
Inhibition of GC activity by XTP in membrane preparations of HEK293 cells stably overexpressing pGC-A. Membranes (10 µg protein per tube) was stimulated with 200 µM GTP/Mn^2+^ and 1 µM ANP at 37°C for 5 min and increasing concentrations of XTP/Mn^2+^ (2–1,000 µM). Data were best fitted by using a competitive binding model at one binding site with an IC_50_ of 145.3 ± 1.2 µM. Values based on the means ± SEM of six independent experiments.

To determine accurate kinetic parameters of pGC-A, all further experiments were performed within linear reaction times (5–10 min) and non-depleting substrate conditions (Figs. S1 and S2). Fig. 2 and Table 2 summarize the kinetic parameters obtained from substrate saturation experiments for pGC-A in the presence of Mn^2+^ or Mg^2+^. In the presence of Mn^2+^, a very robust GC activity with a s_0.5_ of 231 µM and a V_max_ of 9.8 nmol min^−1^ mg^−1^ was observed. The Hill coefficient (*n*_Hill_) of 1.5 indicated strong cooperativity. When Mn^2+^ was replaced by Mg^2+^, GC activity could only be detected when ATP/Mg^2+^ was added. Under these conditions, affinity and cooperativity towards GTP were slightly decreased and V_max_ was reduced to 7.3 nmol min^−1^ mg^−1^. Besides GC activity, an inosinyl cyclase (IC) activity could also be detected. In the presence of Mn^2+^, V_max_, s_0.5_, and *n*_Hill_ of IC activity were within the same range (14.4 nmol min^−1^ mg^−1^, 232 µM, and 1.8, respectively) as for GTP. With Mg^2+^, IC activity strongly decreased to a V_max_ of 1.0 nmol min^−1^ mg^ 1^ and affinity decreased. However, Hill coefficient hardly changed after cofactor substitution. In the presence of Mn^2+^, we could also detect very low V_max_ values for adenylyl (AC) and xanthosinyl cyclase (XC) activities with ∼0.2 nmol min^−1^ mg^−1^. ATP and XTP showed nearly the same affinities towards pGC-A as GTP with a s_0.5_ of 224 and 333 µM, respectively. AC activity showed only a small cooperativity with *n*_Hill_ of 1.1. XTP was best fitted using a substrate inhibition model. However, determination of K_i_ was ambiguous. pGC-A is regulated by ATP decreasing the s_0.5_ of the cyclase [4], [22], [23]. For XTP, no regulation of pGC-A has yet been described. Therefore, we analyzed the influence of XTP on GC activity. Fig. 3 shows that in contrast to ATP, XTP reduced GC activity with an IC_50_ of 145.3±1.2 µM. A simultaneous generation of cXMP was not observed.

**Table 2:** Kinetic parameters of membrane preparations of HEK293 stably overexpressing pGC-A for various nucleotides.

		ATP	CTP	GTP	ITP	TTP	UTP	XTP
Mn^2+^	s_0.5_ [µM]	223.8±17.4	nd	231.4±19.1	231.6±12.3	nd	nd	333.0±58.0
	V_max_ [nmol min^−1^ mg^−1^]	0.18±0.01	nd	9.8±0.3	14.4±0.2	nd	nd	0.21±0.02
	*n*_Hill_	1.1±0.1	–	1.5±0.1	1.8±0.1	–	–	nq
Mg^2+^	s_0.5_ [µM]	nd	nd	271.6±15.8*	1,261±58.7	nd	nd	nd
	V_max_ [nmol min^−1^ mg^−1^]	nd	nd	7.3±0.1*	1.0±0.03	nd	nd	nd
	*n*_Hill_	–	–	1.2±0.1*	2.1±0.2	–	–	–

NC activities were analyzed as mentioned in Materials and Methods. Membranes from HEK293 cells overexpressing pGC-A (10–80 µg of protein per tube) were incubated with 2–7,500 µM (XTP/Me^2+^: 2–2,000 µM) NTP/Me^2+^ in the presence of 1 µM ANP at 37°C for 5–10 min depending on the analyzed NTP. Apparent s_0.5_, V_max_, and *n*_Hill_ represent the means ± SEM of six independent experiments shown in Figs. 1 and 2 and are given in alphabetical order of NTPs. Curves were analyzed by nonlinear regression using Prism version 5.0. nd: not detected, nq: not quantified.

* only detectable in the presence of 500 µM ATP/Mg^2+^.

### Analysis of NC Activity of pGC-A in Intact HEK293 Cells

**Figure 4:**
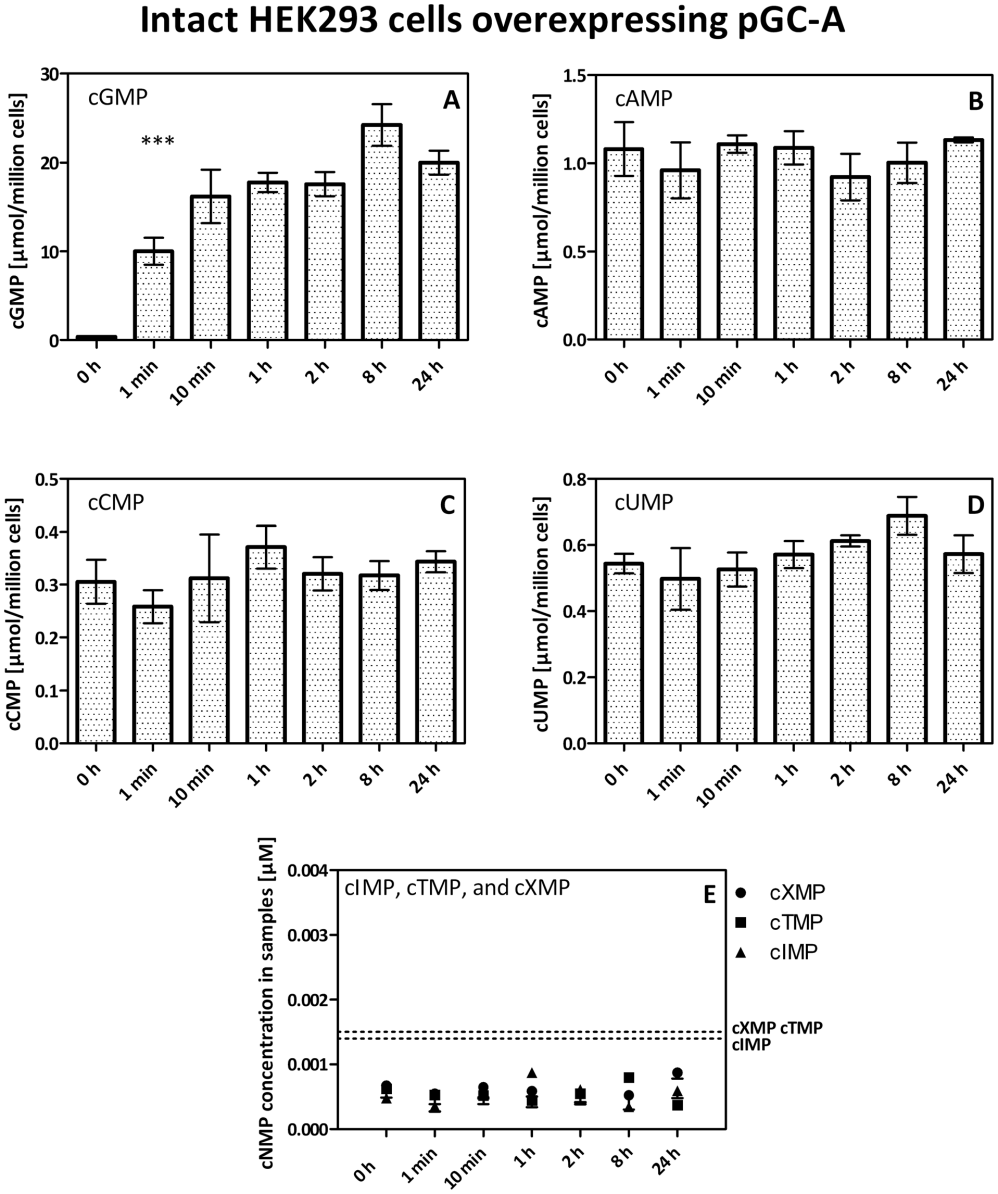
cNMP levels in intact HEK293 cells stably overexpressing pGC-A following stimulation with ANP. HEK293 cells stably overexpressing pGC-A were seeded in 6-well plates for 24 h with 5⋅10^5^ cells per well and stimulated with 1 µM ANP for defined times 10 min after preincubation with IBMX (100 µM). Values of plot A-E are given by means ± SEM of 3–6 independent experiments in µmol/million cells. Please note the different scales of the y-axes in these panels. Plot E: Data points are due to background noise. Dotted lines: lower limit of detection of cIMP, cTMP, and cXMP. ***: p-value ≤0.001.

We also analyzed the formation of cyclic nucleotides by pGC-A after stimulation by ANP (1 µM) in intact HEK293 cells. Stimulation of intact HEK293 cells stably overexpressing rat pGC-A by ANP resulted in a very strong and long-lasting increase of cGMP-formation (Fig. 4A). Interestingly, substantial basal concentrations of cAMP, cUMP, and cCMP were observed in intact HEK293 cells (Figs. 4B–4D). However, stimulation by ANP did not increase cCMP, cUMP and cAMP concentrations in intact HEK293 cells. cXMP, cIMP and cTMP were below the detection limit under basal conditions in intact HEK293 cells and were not increased above the detection limit after ANP stimulation (Fig. 4E).

### Analysis of pGC-E and pGC-F

**Figure 5:**
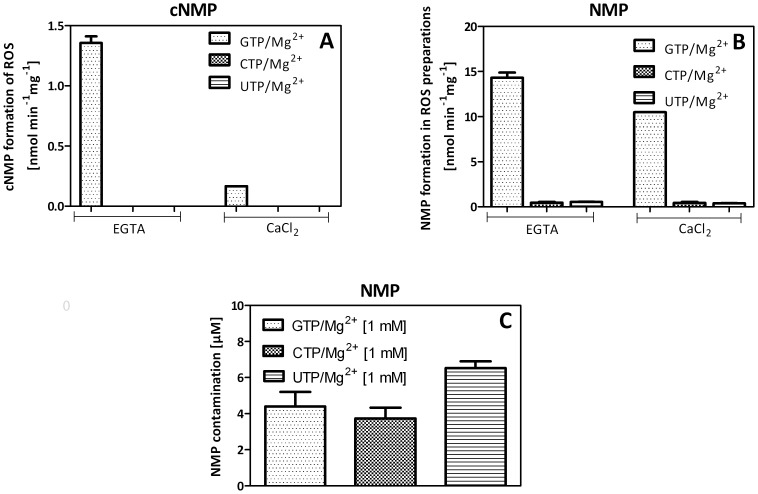
Analysis of NC activity of ROS membrane preparations. Membranes (81 µg rhodopsin per tube) were incubated for 5 min at 30°C with 3.5 mM MgCl_2_ and 1 mM GTP/Mg^2+^, UTP/Mg^2+^ and CTP/Mg^2+^, respectively, and 2 mM EGTA or 2 mM CaCl_2_, as indicated. Reactions were stopped by heating at 95°C for 10 min and analyzed as described in Materials and Methods. Values represent the mean with range of 2–3 independent experiments. Please note the different scales of the y-axes of all panels. A, cNMP formation with membranes; B, NMP formation with membranes; C, control experiments showing contamination of NTP solutions with NMPs.

To answer the question whether the lack of pyrimidinyl cyclase activity of GC in the presence of Mg^2+^ is a general property of GCs, we analyzed preparations of ROS containing pGC-E and pGC-F. Membranes (81 µg rhodopsin per tube) were incubated for 5 min at 30°C with 3.5 mM MgCl_2_ and 1 mM GTP/Mg^2+^, UTP/Mg^2+^, and CTP/Mg^2+^, respectively. As expected, we observed GC activity in samples containing GTP (Fig. 5A). Samples containing whole ROS as well as the addition of CaCl_2_ resulted in a decrease to basal cGMP concentrations. No pyrimidinyl cyclase activity was observed in ROS membranes (Fig. 5A). Besides generation of cGMP, high amounts of nucleoside 5′-monophosphates (NMPs) were detected (Fig. 5B) although a PDE-inhibitor was added. To evaluate if these NMPs were generated by nucleotidases or phosphatases or were contaminants of the stock solution we determined NMP levels in the absence of membrane. As illustrated in Fig. 5C the detection of GMP in Fig. 5B was largely due to enzymatic degradation of GTP. However, the presence of CMP and UMP was due to impurity of NTP standards with about 0.4%.

### NC Activity of CyaA and EF

A previous study on the substrate-specificities of EF and CyaA used radiometric- and HPLC-based assays and focused on Mn^2+^ as cofactor for catalysis [11]. Moreover, complete kinetic analysis was only performed for ATP, CTP and GTP as substrates. In our present study, we used a highly sensitive HPLC-MS/MS-based method and assessed complete kinetics under Mg^2+^ conditions for ATP, CTP, UTP, ITP, GTP, TTP and XTP (Table 3 and Figs. S4 and S5). All NTPs were used as NTP/Mg^2+^ complexes. Thus, the present data complement the previously published data set on NTP/Mn^2+^ complexes. For CyaA the following descending order of k_cat_-values was observed: cAMP>>>> cCMP ≈ cXMP>> cUMP>> cIMP>> cGMP. For EF, k_cat_-values were: cAMP>>>> cCMP>>> cUMP>> cIMP ≈ cGMP>> cTMP ≈ cXMP. For CyaA, highest affinity was observed for TTP followed by XTP>> ATP ≈ GTP> ITP> CTP> UTP. EF exhibited highest affinity for ATP followed by GTP> CTP> ITP> UTP>>>> XTP. For XTP at CyaA and EF, mixed substrate/inhibitor properties were found. At higher concentrations, TTP also exhibited inhibitory properties at CyaA.

**Table 3:** Kinetic data of substrate saturation experiments with CyaA-N and EF.

	CyaA-N		EF	
	K_M_ (µM)	k_cat_ (s^−1^)	K_M_ (µM)	k_cat_ (s^−1^)
AC	171±22.8	1156±50	78.1±8.7	524±14
CC	475±139	3.83±0.54	367±116	1.95±0.26
UC	599±59	0.17±0.009	792±299	0.31±0.07
IC	329±44	0.043±0.002	507±108	0.065±0.007
GC	191±25	0.043±0.002	85±13	0.0024±9E-5
TC	19±5	0.008±0.0003	–	–
	K_i_ (µM) = 1332±365*			
XC	37±7	0.0077±0.0007	161,700±5.3E7^a^	1.02±335^a^
	K_i_ (µM) = 309±57*		K_i_ (µM) = 0.03±8a, *	

a data-fit ambiguous,

* K_i_ for substrate inhibition; N = 3–6; analysis of the data shown in Figs. S4 and S5. AC, adenylyl cyclase; CC, cytidylyl cyclase; GC, guanylyl cyclase; IC, inosityl cyclase; TC, thymidinyl cyclase; UC, uridylyl cyclase; XC, xanthosinyl cyclase.

## Discussion

### NC Activity of Pgcs

sGC possesses purinyl and pyrimidinyl cyclase activity [9]. Garbers and coworkers reported that sea urchin sperm pGC generates cIMP in the presence of Mn^2+^ [6], but kinetic studies were not conducted. Waldman *et al.* studied rat kidney membranes in the presence of Mg^2+^ and found that ATP is no pGC substrate [24]. Lack of generation of cCMP, cUMP, and cAMP was reported using purified preparations of the catalytic core of pGC-A in the presence of Mn^2+^ [7]. However, previous studies were performed by radiometric assays that are not unequivocal in terms of chemical identification of molecules [7], [21], [24]. Here, for the first time, a systematic analysis of NC activity of rat pGC-A was performed using structure-based identification and quantitation by HPLC-MS/MS. Using Mg^2+^, we could confirm that pGC-A lacks AC activity [25] and that substrate specificity of pGC with respect to NTPs (2′-deoxy-NTPs were not studied here) is restricted to GTP and ITP. This is most likely due to the close structural similarity of ITP and GTP. The exchange of Mn^2+^ to Mg^2+^ dramatically decreased formation of cIMP by pGC-A. In contrast, the exchange of Mn^2+^ against Mg^2+^ did not have dramatic negative effects on V_max_ of rat sGC α_1_β_1_ with ITP [9]. In order to understand these striking biochemical biochemical differences, at a molecular level it will be necessary to resolve the crystal structures of both pGC-A and rat sGC α_1_β_1_ in complex with ITP analogs that are not NC substrates. A possible ITP analog for such future crystallization studies is 2′(3′)-*O*-(N-methylanthraniloyl) inosine 5′-triphosphate since 2′(3′)-*O*-(N-methylanthraniloyl)-substituted NTPs bind to various NCs with high affinity [11], [13], [14].

We noted that the catalytic activity of pGC-A in time course experiments was linear with GTP/Mn^2+^ for up to 100 min, whereas for all other NTP/Mn^2+^ conditions and for all NTP/Mg^2+^ conditions, catalysis was linear only for much shorter periods of time (Figs. S1 and S2). Accordingly, incubation time for kinetic studies (Fig. 2) were adjusted to short incubation times, ensuring linearity. Similar to pGC-A, we observed only short linear reaction rates for many substrate conditions at sGC [9]. These data cannot be explained by substrate depletion since the maximum substrate conversion rates were just 8%. Probably, the substrates differ from each other in their ability to stabilize sGC and pGC, GTP being the most effective NTP in this respect [9].

Since no crystallographic data of pGC-A have been reported yet, current models of the structure of pGC-A are derived from homology modeling based on the crystal structure of ACs [10]. pGC is suggested to contain two catalytic domains that act cooperatively [1]. However, a new model postulates that pGCs are asymmetric homodimers with an allosteric and a catalytic domain that bind GTP and ATP, respectively [4]. Kinetics of pGC-A activity are complex [4], [10], [26]–[32]. In the presence of Mn^2+^, V_max_ values of 300 pmol min^−1^ mg^−1^ for bovine adrenal cortex [31] and 19 µmol min^−1^ mg^−1^ for highly purified pGC-A [10] are reported. Values of s_0.5_ range between 0.017 mM for highly purified receptor [10] and 0.2 mM for rat tissue preparations [32]. The Hill coefficients (*n*_Hill_) range between 1.4−1.74 indicating a highly cooperative behavior [6], [26]–[32]. In the presence of Mg^2+^ V_max_ values were between 0.069 nmol min^−1^ mg^−1^ for rat lung [27], 1.1 nmol min^−1^ mg^−1^ for HEK293 cells stably expressing pGC-A [23], and 28 µmol min^ 1^ mg^−1^ for purified rat pGC-A expressed in *Sf*9 cells [10]. The Hill coefficients range between 1.2–1.4 [10], [32] and s_0.5_ values were between 0.37 mM for highly purified receptor [10] and 0.73 mM for membrane preparations of HEK293 cells stably overexpressing pGC-A [23]. The s_0.5_ and V_max_ values as well as Hill slopes of this study are in good agreement with literature data. Using sea urchin sperm membranes, Garbers *et al*. reported an s_0.5_ of 0.3 mM for ITP/Mn^2+^ with a concave reciprocal plot indicating cooperative behavior [6]. We could confirm cooperativity with *n*_Hill_ of 1.8 for IC activity and s_0.5_ was within the reported range (232 µM). IC activity could also been detected for the first time in the presence of Mg^2+^. Here, V_max_ value was significantly reduced to 1 nmol min^−1^ mg^−1^ and s_0.5_ increased to the millimolar concentration range.

We show for the first time that pGC-A generates cAMP in the presence of Mn^2+^. Although V_max_ values were fifty-fold lower than for GTP, affinities were in the same range as GTP. Very recently, ATP has been shown to bind at an allosteric site in the catalytic domain reducing Hill coefficient and K_M_ [4]. Moreover, ATP inhibits the catalysis of pGC-A in a competitive manner [21]. This observation was very surprising for the authors because ATP is reported to activate full-length pGC-A [21]. Here, we show that this discrepancy is due to the AC activity of pGC-A. We also found that XTP is a substrate of pGC-A in the presence of Mn^2+^. We observed a s_0.5_ within the range of GTP and a substrate inhibition at millimolar concentrations indicating that XTP influences GC activity. Indeed, we observed an inhibitory effect of XTP on GC activity in the presence of Mn^2+^. In contrast to sGC that shows pyrimidinyl cyclase activity with Mn^2+^ as cofactor [9], pGC-A did not accept CTP, UTP and TTP as substrates. Our present data with holo-pGC-A regarding UTP and CTP are in accord with previous data on the catalytic core of pGC-A [7], [21]. The pyrimidinyl cyclase activity of sGC may be due to unique structural features of the enzyme [33]. A molecular explanation for these biochemical differences between pGC-A and sGC hinges on the resolution of crystal structures.

The focus of our present study was the analysis of the substrate-specificity of wild-type pGC-A. The precise elucidation of the molecular basis for the complex kinetic properties of pGC-A and the analysis of the interaction of the catalytic and allosteric site will require extensive mutagenesis studies [4], . As an additional approach, (*Rp*)- and (*Sp*)-diastereoisomers of nucleoside 5′-*O*-(1- and 2-thio)triphosphates should be studied since the stereoisomers differ from each other in their ability to serve as substrates for pGCs [36].

Dessauer *et al.* [37] reported that PP_i_, one of the products of the NC reaction, at concentrations between 0.3–1.6 mM, can inhibit catalysis of mammalian membranous AC. Hence, we have to consider the possibility that some of the biphasic kinetics observed with pGC-A in the presence of ITP/Mg^2+^ and XTP/Mn^2+^ (Figs. 2D and F) are due to inhibition of catalysis by PP_i_. This is unlikely because inhibitory effects would have been expected at high catalytic rates, resulting in high concentrations of PP_i_. However, XTP/Mn^2+^ was only a poor substrate, and the biphasic component with ITP/Mg^2+^ was observed with low substrate concentrations. Moreover, enzyme kinetics with highly efficient substrates, GTP/Mn^2+^ and ITP/Mn^2+^, were monophasic (Figs. 2A and B). Furthermore, in the time course experiments, non-linear reaction rates were observed with several efficient and inefficient substrates but not the efficient substrate GTP/Mn^2+^. Thus, there is no correlation between non-linear kinetics and high catalysis rates. Sophisticated studies with (*Rp*)- and (*Sp*)-diastereoisomers of nucleoside 5′-*O*-(1- and 2-thio)triphosphates [36] also argue against the hypothesis that inhibition of catalysis by PP_i_ plays a major role for complex enzyme kinetics of pGC.

Stimulation of intact HEK293 cells stably overexpressing pGC-A with ANP resulted in a massive and long-lasting increase of cGMP formation (Fig. 4A), whereas no increases in cAMP, cIMP, cXMP, cUMP, cCMP and cTMP were detected. Our failure to detect increases in cNMP concentrations in intact cells other than cGMP is unlikely due to cNMP degradation because we conducted experiments in the presence of a non-selective phosphodiesterase inhibitor. Most likely, in intact cells only very low levels of ITP and XTP are present [38]. Moreover, AC activity of pGC-A was very low, too, so that putative cAMP increases could not be detected. We could not confirm the natural existence of cTMP and cXMP in cells (Fig. 4E), although cTMP and cXMP had been tentatively identified in rat tissue [39]. Probably, the previously reported detection of cTMP and cXMP in tissues is an artifact due to insufficient sensitivity of the previously available mass spectrometers [40].

In ROS membrane preparations expressing pGC-E and pGC-F we observed GC activities that are typical for incubations at different Ca^2+^-concentrations and using GTP/Mg^2+^ as substrate (Fig. 5A). For example, chelating Ca^2+^ by EGTA resulted in about eight-fold higher activity than keeping the Ca^2+^-concentration high. These results are consistent with previous reports on GC activation profiles in bovine ROS reviewed in Ref. 5. All GCs studied so far, i.e. sGC [9], and pGC A, pGC-E and pGC-F share the property that in the presence of Mg^2+^ they lack pyrimidinyl cyclase activity (Figs. S1 and S2 and 5).

### NC Activity of CyaA and EF

The exotoxins CyaA and EF have already been demonstrated to generate cIMP, cCMP and cUMP [11], [12] and are potently inhibited by various purine and pyrimidine nucleotide analogs [13], [14]. In contrast to sGC and pGC, broad substrate specificities of CyaA and EF were even detected in presence of Mg^2+^ (Table 3) In fact, both CyaA and EF exhibited CC activity, but k_cat_ values were approximately 300 times lower than for AC activity. Both toxins also produced cUMP, cGMP, cIMP and cXMP at very low catalytic rates. cTMP production could only be detected for CyaA. XTP and TTP both exhibited substrate inhibition kinetics. Thus, among all NCs studied so far, CyaA and EF possess the broadest substrate-specificity. This is most evident in the presence of Mg^2+^.

### Conclusions and Future Studies

In contrast to sGC [9], pGC-A lacks pyrimidinyl cyclase activity. Since GTP and ATP have already been shown to bind at the allosteric binding domain of pGC-A [4] future studies have to investigate whether further nucleotides regulate pGC-A by binding at this domain. sGC-generated cCMP and cUMP may serve a unique signal transduction role like cCMP and cUMP produced by the bacterial “adenylyl” cyclase toxins edema factor from *Bacillus anthracis* and CyaA from *Bordetella pertussis* [9], [11], [41].

UC- and CC activity of certain NCs cannot be considered as trivial substrate leakiness of enzymes since some NCs such as the pGCs studied herein, possess a higher substrate-specificity than sGC, EF and CyaA. Future studies will have to assess the substrate-specificities of membranous and soluble ACs. At least with regard to inhibitors, these enzymes also exhibit broad base-specificity like sGC, EF and CyaA [13], [42]. Moreover, we have not yet studied pGCB, pGC-C and pGC-D [1]. At the time being, we have no evidence for the existence of a mammalian NC with preferential CC- and/or UC activity [43]. It remains to be determined whether the quite considerable basal levels of cUMP and cCMP serendipitously observed in HEK293 cells (Figs. 4C and 4D) are due to the activity of sGC or an AC. We can at least exclude pGC-A as a regulator of cellular cCMP and cUMP levels. Based on the broad substrate-specificity of EF and CyaA demonstrated in our previous study [11] and herein, it is worthwhile to analyze the substrate specificity of other bacterial NCs. ExoY from *Pseudomonas aeruginosa*, structurally related to EF and CyaA, is an excellent candidate [44], [45]. Specifically, ExoY was previously classified as AC, but a recent study revealed that the toxin possesses much higher GC- than AC activity [45]. A tentative CC- and UC activity of ExoY has still to be explored.

## Acknowledgments

We thank Mrs. Annette Garbe, Mrs. Ingelore Hackbarth, Mrs. Juliane von der Ohe, and Mrs. Petra Behnen for expert technical assistance. We thank Dr. Michaela Kuhn (Institute of Cardiovascular Physiology, University of Würzburg, Germany) for providing HEK pGC-A cells. Thanks are also due to the reviewers for their helpful comments.

## Supporting Information

Figure S1 **Time-courses of NC activity in membrane preparations of HEK293 cells stably overexpressing pGC-A.** Membranes (10–50 µg of protein) were incubated for 2–90 min at 37°C in the presence of 200 µM NTP/Mn^2+^. Values represent the means ± SEM of 3–6 independent experiments. Data points of plot E and F are due to background noise. Please note the different scales of the y-axes in all panels. Dotted lines: lower limit of detection.
(TIF)

Figure S2 **A and B: Time-courses of NC activity in membrane preparations of HEK293 cells stably overexpressing pGC-A.** Membranes (10–80 µg of protein) were incubated for defined times at 37°C in the presence of 200 µM NTP/Mg^2+^ as indicated. Values represent the mean ± SEM of six independent experiments. Please note the different scales of the y-axes in both panels. *: only detectable in the presence of 500 µM AT/Mg^2+^P. Figure S2C: Analysis of cNMP formation in the presence of Mg^2+^. Ten µg of protein were incubated for 60 min at 37°C in the presence of 200 µM NTP/Mg^2+^ as indicated. Dotted lines: lower limit of detection. Values represent the mean ± SEM of three independent experiments.
(TIF)

Figure S3 **Substrate specificity of pGC-A in the presence of Triton X-100.** Membrane preparations of HEK293 cells stably overexpressing pGC-A (100 µg of protein per tube) were incubated for 5 min at 37°C in the presence of 200 µM NTP/Mg^2+^ and 0.1% (m/v) Triton X-100. Values represent the mean ± SEM of three independent experiments.
(TIF)

Figure S4 **Calibration curves of cNMPs and corresponding substrate saturation experiments with CyaA-N and EF, respectively.** A-C cCMP calibration and CTP saturation, D-F cAMP calibration and ATP saturation, G-I cGMP calibration and GTP saturation and J-L cUMP calibration and UTP saturation. For kinetic parameters see Table 3.
(TIF)

Figure S5 **Calibration curves of cNMPs and corresponding substrate saturation experiments with CyaA-N and EF, respectively.** A-C cIMP calibration and ITP saturation, D-F cXMP calibration and XTP saturation, G-H cTMP calibration and TTP saturation. cTMP formation was only detected in CyaA-N but not in EF. For kinetic parameters see Table 3.
(TIF)
