# Supplementary figures and images for: Nucleotidyl Cyclase Activity of Particulate Guanylyl Cyclase A: Comparison with Particulate Guanylyl Cyclases E and F, Soluble Guanylyl Cyclase and Bacterial Adenylyl Cyclases Cyaa and Edema Factor
Source: PLoS One. 2013 Jul 29;8(7):e70223. doi: 10.1371/journal.pone.0070223 (PMC3726482; doi:10.1371/journal.pone.0070223)

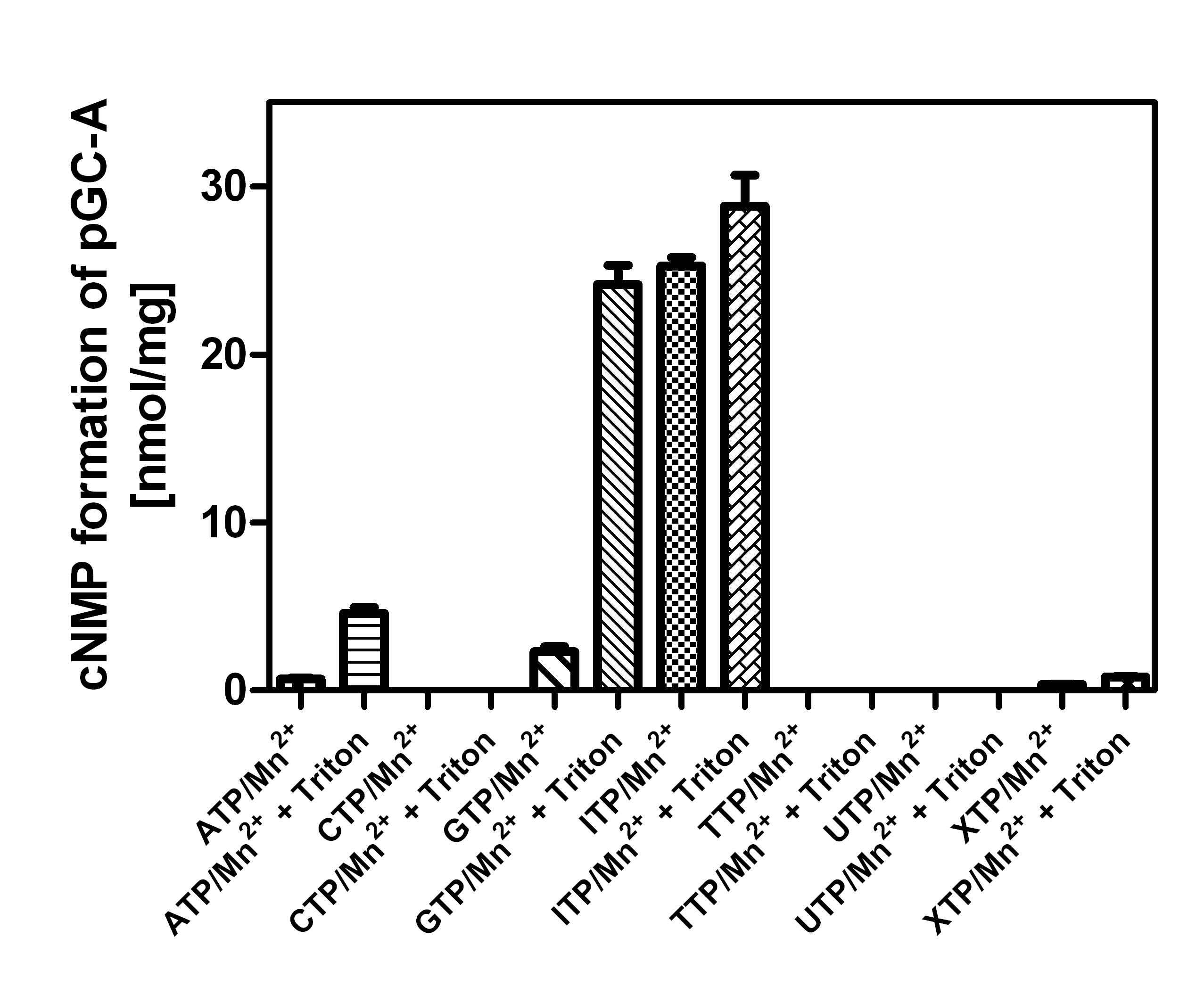

Supplement: Figure S3 — Substrate specificity of pGC-A in the presence of Triton X-100. Membrane preparations of HEK293 cells stably overexpressing pGC-A (100 µg of protein per tube) were incubated for 5 min at 37°C in the presence of 200 µM NTP/Mg2+ and 0.1% (m/v) Triton X-100. Values represent the mean ± SEM of three independent experiments. (TIF) [file pone.0070223.s003.tif]
